# Effect of Wine and Vinegar Processing of *Rhizoma Corydalis* on the Tissue Distribution of Tetrahydropalmatine, Protopine and Dehydrocorydaline in Rats

**DOI:** 10.3390/molecules17010951

**Published:** 2012-01-18

**Authors:** Zhiying Dou, Kefeng Li, Ping Wang, Liu Cao

**Affiliations:** 1 College of Chinese Materia Medica, Tianjin University of Traditional Chinese Medicine, Tianjin 300193, China; 2 Department of Biological Sciences, Michigan Technological University, Houghton, MI 49931, USA; Email: kefengl@mtu.edu

**Keywords:** *Rhizoma Corydalis*, processing, HPLC, tissue distribution

## Abstract

Vinegar and wine processing of medicinal plants are two traditional pharmaceutical techniques which have been used for thousands of years in China. Tetrahydropalmatine (THP), dehydrocorydaline (DHC) and protopine are three major bioactive molecules in *Rhizoma Corydalis.* In this study, a simple and reliable HPLC method was developed for simultaneous analysis of THP, DHC and protopine in rat tissues after gastric gavage administration of *Rhizoma Corydalis*. The validated HPLC method was successfully applied to investigate the effect of wine and vinegar processing on the compounds’ distribution in rat tissues. Our results showed that processing mainly affect the T_max_ and mean residence time (MRT) of the molecules without changing their C_max_and AUC_0–24__ h_ Vinegar processing significantly increased the T_max_ of DHC in heart, kidney, cerebrum, cerebrellum, brain stem and striatum and prolonged theT_max_ of protopine in brain. No significant changes were observed on the T_max_ of THP in rat tissues after vinegar processing. Wine processing reduced the T_max_of protopine and DHC in liver and spleen and T_max_ of protopine in lung, but increased the T_max_ of THP in all the rat tissues examined. To our knowledge, this is the first report on the effects of processing on the tissue distribution of the bioactive molecules from *Rhizoma Corydalis*.

## 1. Introduction

**Figure 1:**
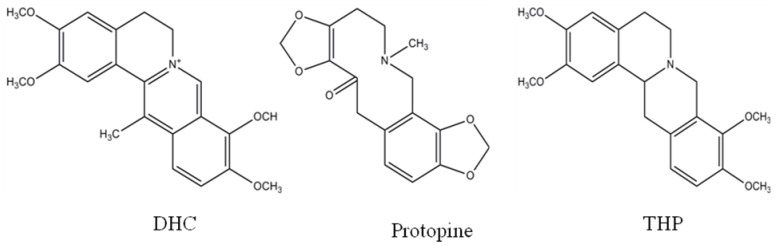
Chemical structures of dehyrocorydaline (DHC), protopine and tetrahydropalmatine (THP).

The dried tuber of *Corydalis yanhusuo* W.T. Wang (*Rhizoma Corydalis*) has been successfully and regularly used in some Asian countries to alleviate various painful symptoms including spastic, abdominal and menstrual for hundreds of years [1]. The major bioactive components are three alkaloid compounds, namely tetrahydropalmatine (THP), dehydrocorydaline (DHC) and protopine [2,3] (Figure 1). Regulation of D_2_ dopamine receptors in central nervous system by THP and its analogues is the main analgesic mechanism [4]. Recently, it was found that *Rhizoma Corydalis* has anti-inflammatory and anti-tumor activities by suppressing IL-8 secretion, inhibiting aromatase activity or reducing cytochrome c release [5,6,7]. *Rhizoma Corydalis* can also promote blood circulation and is being used for the treatment of coronary heart diseases such as myocardial ischemia, infarction and stunning [8]. The anti-ischemia effect of *Rhizoma Corydalis* is associated with the direct protective function of DHC on cardiomyocytes and the inhibition of myocardial apoptosis [9,10]. The alkaloid protopine in *Rhizoma Corydalis* is known to have vasodilator effect [11].

Previous reports have described the HPLC methods for simultaneous determination of THP and protopine in *Rhizoma Corydalis* and rat plasma after administration of *Rhizoma Corydalis* [12,13]. A HPLC-MS/MS method was also developed for quantitative determination of ten alkaloids in methanol and ethyl acetate extract of *Rhizoma Corydalis* [2]. However, there is no report on the simultaneous determination of the main bioactive compounds in rat tissues after administration of the ethanol extract of *Rhizoma Corydalis*.

Medicinal plants sometimes require specific processing steps such as drying, water processing (steaming and roasting) and fire processing (stir-frying with wine, vinegar, salt or honey) to enhance the efficacy or reduce the toxicity of their components [14]. The processing of *Rhizoma Corydalis* using wine and vinegar was first documented in Lei's Treatise on Processing of Drugs (*Leigong Pao Zhi Lun* in Chinese) in the Tang Dynasty of China (618–907 AD). Nowadays, the procedures for processing *Rhizoma Corydalis* using vinegar and wine have been standardized according to pharmacists’ experiences and are listed in Pharmacopoeia of Chinese Medicine and the National Guideline of Traditional Chinese Medicinal Plants Processing, respectively [15]. 

The mechanisms of wine and vinegar processing have not yet been fully elucidated chemically and pharmacologically [16]. Comparison of analgesic and anti-inflammatory effects of wine and vinegar processed products of *Rhizoma Corydalis* showed that vinegar processed products are better than wine processed products [17]. Our previous results showed that the content of THP, DHC and protopine in ethanol extract of both wine and vinegar processed *Rhizoma Corydalis* was higher than that in unprocessed control [18]. In addition, vinegar and wine might form a chelate complex with the bioactive compounds in the medicinal plants and therefore change the pharmacokinetic action of the compounds. However, there is little information on the changes of the distribution of the bioactive compounds in rat tissues after the processing of the medicinal plants. The objective of this study was to develop a simple and accurate HPLC method for simultaneous determination of THP, DHC and protopine in rat tissues and investigate the effect of wine and vinegar processing of *Rhizoma Corydalis* on the distribution of THP, DHC and protopine in rat tissues.

## 2. Results and Discussion

### 2.1. Results

#### 2.1.1. Method Validation

**Figure 2:**
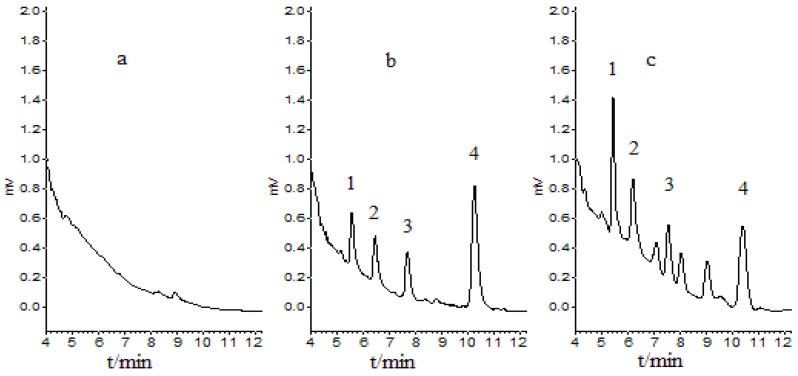
Representative chromatographs of (**a**) a blank liver sample; (**b**) a liver sample spiked with analytes (THP (0.306µg/mL), DHC (0.294 µg/mL), protopine (0.320 µg/mL) and nuciferine (1.0 µg/mL); (**c**) A liver sample after administration of vinegar processed *Rhizoma Corydalis*. 1-protopine; 2-THP; 3-nuciferine; 4-DHC.

An analytical method for the simultaneous determination of THP, DHC and protopine in rat tissues by HPLC-UV was developed and validated. Figure 2 shows the representative chromatographs of a liver sample. The retention time was about 5.1 min for protopine, 6.1 min for THP, 7.4 min for internal standard nuciferine and 10.2 min for DHC. No interfering peaks were observed in drug-free tissue at the retention time of the analytes.

The regression equations, correlation coefficients, linear ranges are listed in Table 1. The correlation coefficient (r^2^) for each calibration curve was over 0.99, which indicated that there was excellent correlation between the ratio of peak area and concentration for each compound within the linear range. The intra- and inter-day precision values (R.S.D.%) of THP, DHC and protopine in different tissue samples were all less than 5.83%. The relative extraction recoveries of THP and DHC ranged from 94.77%~105.82% and from 93.54%~104.76%. The relative recovery of protopine was from 92.19% to 103.91%. This indicated that the method is reproducible and reliable for quantitative analysis of THP, DHC and protopine in rat tissue samples simultaneously and the extraction recovery was consistent and reproducible.

**Table 1:** Calibration curve of THP, DHC and protopine in rat tissues. Y: Peak area ratios of analytes to internal standard; X: Concentration of analytes in rat tissues.

Tissues	Compounds	Regression equation	Correlation coefficient (r^2^)	Linear range (µg/mL)
Heart	THP	Y = 2.1542X + 0.0544	0.9997	0.0306~0.612
	DHC	Y = 10.686X + 0.0350	0.9997	0.0294~0.588
	Protopine	Y = 2.2532X + 0.0191	0.9996	0.0320~0.640
Liver	THP	Y = 2.7012X + 0.0026	0.9983	0.0306~3.06
	DHC	Y = 13.784X − 0.1516	0.9990	0.0294~2.94
	Protopine	Y = 2.9222X + 0.0080	0.9992	0.0320~3.20
Spleen	THP	Y = 2.6201X + 0.0310	0.9987	0.0306~3.06
	DHC	Y = 12.847X + 0.0475	0.9984	0.0294~2.94
	Protopine	Y = 2.7289X + 0.0966	0.9990	0.0320~3.20
Lung	THP	Y = 2.7128X + 0.0130	0.9980	0.0306~3.06
	DHC	Y = 12.801X + 0.0007	0.9994	0.0294~2.94
	Protopine	Y = 2.8704X − 0.0077	0.9984	0.0320~3.20
Kidney	THP	Y = 2.5418X + 0.0957	0.9981	0.0306~3.06
	DHC	Y = 12.299X + 0.2340	0.9980	0.0294~2.94
	Protopine	Y = 2.6138X + 0.0709	0.9989	0.0320~3.20
Brain	THP	Y = 2.5324X + 0.0217	0.9999	0.0306~0.306
	DHC	Y = 12.657X − 0.0744	0.9998	0.0294~0.294
	Protopine	Y = 2.5671X + 0.0143	0.9997	0.0320~0.320

#### 2.1.2. Effect of Vinegar and Wine Processing on the Tissue Distribution of THP

**Figure 3:**
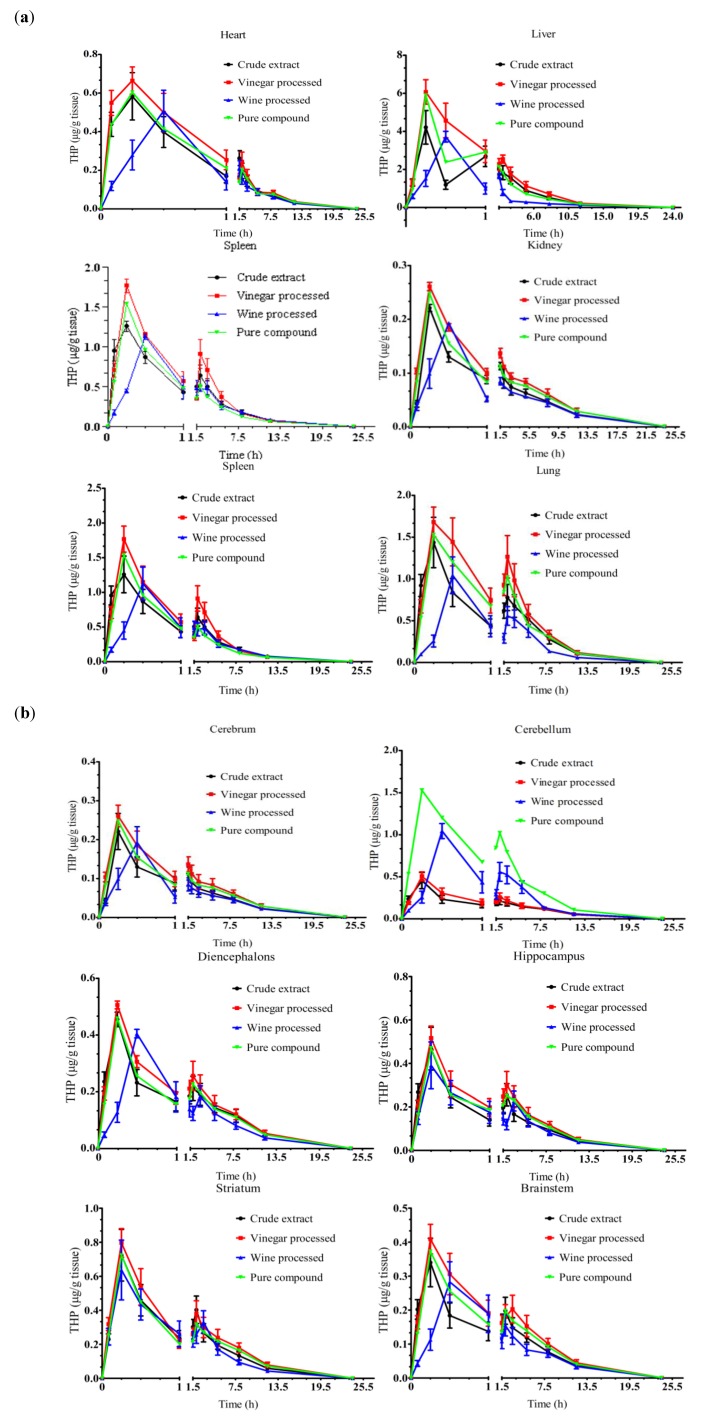
Time dependent changes of THP in rat tissues. (**a**) Heart, liver, spleen, lung and kidney; (**b**) Six parts of brain including cerebrum, cerebellum, diencephalons, brainstem, hippocampus, striatum. Data are mean ± SD (n = 5).

THP first incorporated into the liver and then transported to other tissues (spleen, lung, heart, kidney and brain; Figure 3). The absorption, distribution and elimination of THP were rapid since THP was detected in all the tissues examined within 5 min after administration and was rarely found after 8 h. The concentration of THP reached maxima in heart, liver, spleen, lung, cerebrum, diencephalons and brain stem at 15 min after administration of unprocessed and vinegar-processed *Rhizoma Corydalis*. No significant differences were observed on T_max_ of THP between the vinegar processed and unprocessed group, but THP achieved C_max_ at 30 min after administration of *Rhizoma Corydalis* processed with wine.

There were no significant changes on C_max_ and AUC_0–24 h_ of THP after vinegar and wine processing in all the tissues examined (Table 2). The effect of processing on the mean residence time (MRT) of THP varied among the tissues and between two processing methods. The MRT of THP was notably delayed in heart, spleen, lung, kidney, cerebrum, cerebellum, diencephalons and brainstem as a result of wine processing. However, the MRT of THP with wine processing was shorter in liver than the control groups. Vinegar processing significantly reduced the MRT of THP in spleen and kidney, while, it did not notably change the MRT of THP in other tissues examined. 

**Table 2:** Pharmacokinetic parameters of THP in rat tissues. Data are mean ± SD (n = 5). Data of four groups in each tissue indexed by different letters are significantly different according to one-way ANOVA followed by Tukey’s test (*p* < 0.05).

Tissue	T_max_	C_max_ (µg/mL)	AUC_0–24__ h_ (µg·h/mL)	MRT(h)
**Heart**				
Crude extract	0.25 ± 0.01b	0.59 ± 0.12a	1.44 ± 0.25a	4.61 ± 0.08b
Vinegar processed	0.25 ± 0.03b	0.66 ± 0.07a	1.63 ± 0.28a	4.64 ± 0.11b
Wine processed	0.51 ± 0.01a	0.51 ± 0.11a	1.22 ± 0.25a	5.40 ± 0.19a
Pure compound	0.25 ± 0.00b	0.60 ± 0.18a	1.43 ± 0.32a	4.83 ± 0.13b
**Liver**				
Crude extract	0.25 ± 0.00b	4.22 ± 0.87a	6.91 ± 2.08a	4.52 ± 0.08a
Vinegar processed	0.26 ± 0.02b	6.07 ± 0.64a	6.04 ± 2.87a	4.28 ± 0.07a
Wine processed	0.53 ± 0.04a	3.72 ± 0.78a	5.86 ± 1.22a	3.78 ± 0.13b
Pure compound	0.21 ± 0.01b	7.84 ± 1.74a	5.57 ± 2.60a	3.99 ± 0.08b
**Spleen**				
Crude extract	0.25 ± 0.05b	1.26 ± 0.27a	3.88 ± 0.67a	4.78 ± 0.10b
Vinegar processed	0.27 ± 0.01b	1.77 ± 0.19a	4.71 ± 0.85a	4.23 ± 0.07c
Wine processed	0.52 ± 0.04a	1.13 ± 0.24a	3.53 ± 0.71a	6.21 ± 0.24a
Pure compound	0.23 ± 0.00b	1.54 ± 0.45a	3.27 ± 0.74a	4.34 ± 0.09c
**Lung**				
Crude extract	0.25 ± 0.01b	1.44 ± 0.30a	5.33 ± 0.91a	5.74 ± 0.13b
Vinegar processed	0.28 ± 0.01b	1.68 ± 0.18a	7.02 ± 1.18a	5.27 ± 0.23b
Wine processed	0.51 ± 0.03a	1.04 ± 0.22a	6.56 ± 0.72a	6.23 ± 0.27a
Pure compound	0.26 ± 0.08b	1.53 ± 0.45a	5.93 ± 1.32a	5.34 ± 0.15b
**Kidney**				
Crude extract	0.51 ± 0.00a	1.66 ± 0.33a	4.69 ± 0.83a	5.17 ± 0.13b
Vinegar processed	0.53 ± 0.02a	2.12 ± 0.42a	6.78 ± 1.27a	4.50 ± 0.08c
Wine processed	0.58 ± 0.05a	1.45 ± 0.31a	4.32 ± 0.87a	5.65 ± 0.21a
Pure compound	0.33 ± 0.09b	2.05 ± 0.57a	6.27 ± 1.43a	4.37 ± 0.10c
**Cerebrum**				
Crude extract	0.25 ± 0.01b	0.22 ± 0.046a	0.75 ± 0.13a	7.82 ± 0.26b
Vinegar processed	0.28 ± 0.03b	0.26 ± 0.026a	0.95 ± 0.17a	7.24 ± 0.24b
Wine processed	0.51 ± 0.07a	0.19 ± 0.04a	0.66 ± 0.13a	9.39 ± 0.46a
Pure compound	0.26 ± 0.01b	0.25 ± 0.072a	0.86 ± 0.20a	8.0 ± 0.25b
**Cerebellum**				
Crude extract	0.25 ± 0.02b	0.21 ± 0.04a	0.96 ± 0.16a	7.50 ± 0.23c
Vinegar processed	0.27 ± 0.03b	0.24 ± 0.03a	1.14 ± 0.20a	8.28 ± 0.36bc
Wine processed	0.55 ± 0.01a	0.19 ± 0.04a	0.87 ± 0.17a	8.52 ± 0.46ab
Pure compound	0.26 ± 0.02b	0.15 ± 0.03a	0.99 ± 0.22a	9.20 ± 0.61a
**Diencephalons**				
Crude extract	0.23 ± 0.01b	0.45 ± 0.10a	1.70 ± 0.29a	7.76 ± 0.23b
Vinegar processed	0.25 ± 0.01b	0.51 ± 0.06a	1.88 ± 0.33a	7.31 ± 0.29b
Wine processed	0.52 ± 0.03a	0.40 ± 0.09a	1.38 ± 0.27a	9.06 ± 0.57a
Pure compound	0.27 ± 0.04b	0.46 ± 0.13a	1.67 ± 0.38a	7.70 ± 0.28b
**Brainstem**				
Crude extract	0.25 ± 0.02b	0.34 ± 0.07a	1.33 ± 0.23a	6.82 ± 0.21b
Vinegar processed	0.28 ± 0.01b	0.41 ± 0.04a	1.66 ± 0.30a	7.20 ± 0.25b
Wine processed	0.52 ± 0.01a	0.28 ± 0.06a	1.11 ± 0.22a	9.12 ± 0.47a
Pure compound	0.25 ± 0.00b	0.37 ± 0.11a	1.49 ± 0.34a	7.53 ± 0.26b
**Hippocampus**				
Crude extract	0.22 ± 0.03a	0.47 ± 0.10a	1.57 ± 0.27a	6.55 ± 0.20a
Vinegar processed	0.25 ± 0.01a	0.52 ± 0.05a	1.98 ± 0.35a	6.78 ± 0.29a
Wine processed	0.27 ± 0.02a	0.39 ± 0.11a	1.49 ± 0.30a	6.78 ± 0.30a
Pure compound	0.24 ± 0.05a	0.47 ± 0.13a	1.79 ± 0.40a	7.04 ± 0.23a
**Striatum**				
Crude extract	0.25 ± 0.02a	0.72 ± 0.15a	2.40 ± 0.42a	6.30 ± 0.18b
Vinegar processed	0.22 ± 0.07a	0.79 ± 0.09a	2.77 ± 0.49a	6.33 ± 0.28b
Wine processed	0.28 ± 0.01a	0.64 ± 0.18a	2.10 ± 0.42a	5.34 ± 0.17c
Pure compound	0.21 ± 0.03a	0.72 ± 0.21a	2.47 ± 0.57a	7.69 ± 0.26a

#### 2.1.3. Effect of Vinegar and Wine Processing on the Tissue Distribution of DHC

**Figure 4:**
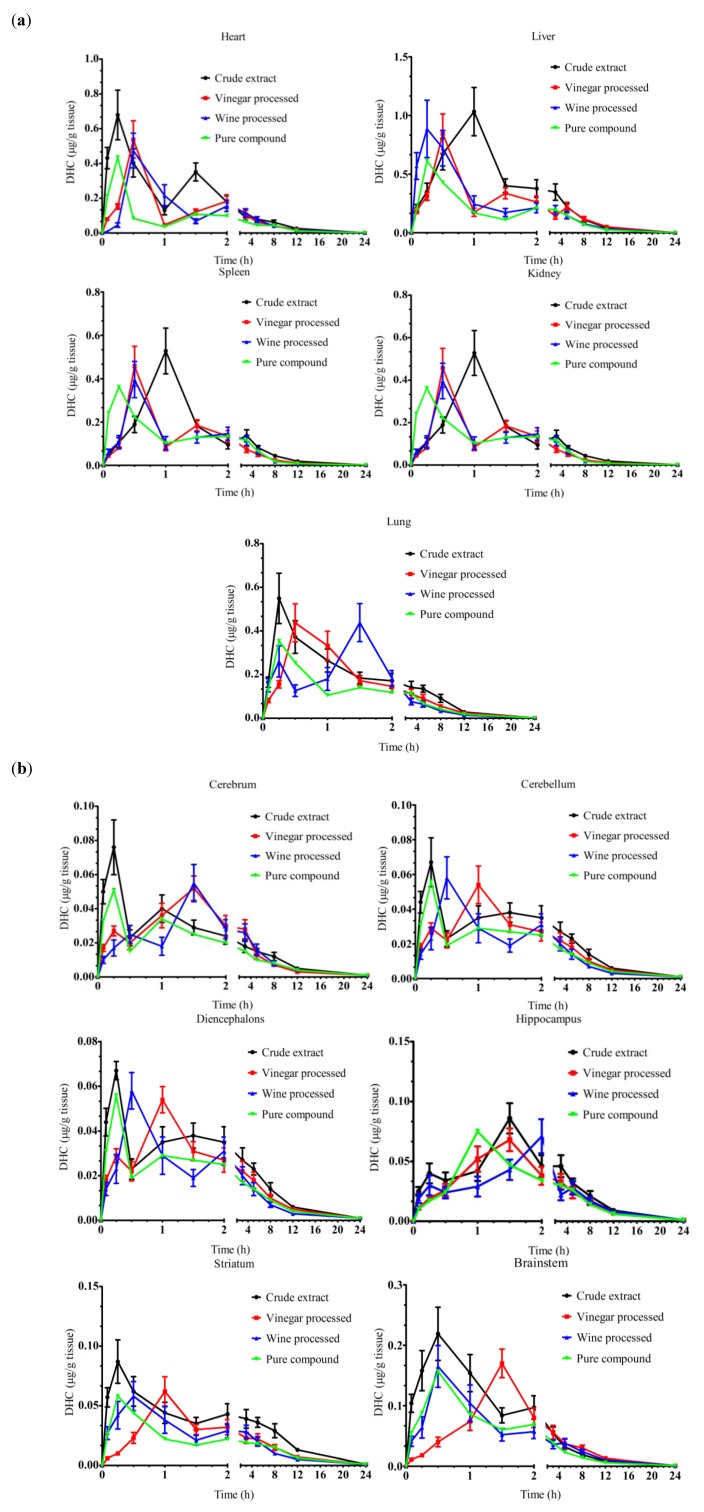
Time dependent changes of DHC in rat tissues. (**a**) Heart, liver, spleen, lung and kidney; (**b**) Six parts of brain including cerebrum, cerebellum, diencephalons, brainstem, hippocampus, striatum. Data are mean ± SD (n = 5).

The time to reach C_max_ for DHC was significantly increased in heart, kidney, cerebrum, brainstem and striatum as a result of vinegar processing (Table 3 and Figure 4). T_max_ of DHC in vinegar processing groups was twice higher in heart and kidney, three times higher in brain stem, four times higher in cerebellum and striatum and six times higher in cerebrum than in the unprocessed control. Wine processing sped up the distribution of DHC in liver, spleen, diencephalons. However, the time to reach the peak concentration was delayed in heart, kidney, cerebrum and striatum after wine processing. Both vinegar and wine processing did not significantly change T_max_ of DHC in lung. 

**Table 3:** Pharmacokinetic parameters of DHC in rat tissues. Data are mean ± SD (n = 5). Data of four groups in each tissue indexed by different letters are significantly different according to one-way ANOVA followed by Tukey’s test (*p* < 0.05).

Tissue	T_max_ (h)	C_max_ (µg/mL)	AUC_0–24__ h_ (µg·h/mL)	MRT (h)
**Heart**				
Crude extract	0.25 ± 0.01b	0.68 ± 0.14a	1.33 ± 0.23a	4.20 ± 0.07a
Vinegar processed	0.51 ± 0.00a	0.54 ± 0.10a	0.97 ± 0.18a	5.05 ± 0.15a
Wine processed	0.53 ± 0.01a	0.47 ± 0.10a	0.99 ± 0.20a	6.12 ± 0.36a
Pure compound	0.25 ± 0.00b	0.44 ± 0.13a	0.85 ± 0.12a	8.72 ± 6.38a
**Liver**				
Crude extract	1.12 ± 0.02a	1.03 ± 0.21a	2.90 ± 0.51a	4.48 ± 0.12bc
Vinegar processed	0.53 ± 0.03b	0.85 ± 0.17a	2.13 ± 0.39ab	6.43 ± 0.20a
Wine processed	0.25 ± 0.00c	0.88 ± 0.25a	1.89 ± 0.40ab	4.19 ± 0.13c
Pure compound	0.25 ± 0.00c	0.62 ± 0.18a	1.57 ± 0.34b	4.85 ± 0.10b
**Spleen**				
Crude extract	1.01 ± 0.07a	0.53 ± 0.10a	1.13 ± 0.20a	5.06 ± 0.10a
Vinegar processed	0.50 ± 0.06b	0.46 ± 0.09a	0.78 ± 0.14a	3.97 ± 0.11b
Wine processed	0.58 ± 0.00b	0.40 ± 0.09a	0.83 ± 0.17a	3.50 ± 0.10c
Pure compound	0.25 ± 0.10c	0.36 ± 0.08a	0.83 ± 0.19a	3.26 ± 0.08c
**Lung**				
Crude extract	0.50 ± 0.00a	0.58 ± 0.12a	1.32 ± 0.23a	5.44 ± 0.15a
Vinegar processed	0.54 ± 0.02a	0.50 ± 0.10a	0.84 ± 0.16ab	4.34 ± 0.11b
Wine processed	0.51 ± 0.09a	0.43 ± 0.10a	0.98 ± 0.20ab	4.61 ± 0.15b
Pure compound	0.25 ± 0.01b	0.37 ± 0.11a	0.57 ± 0.13b	3.98 ± 0.10c
**Kidney**				
Crude extract	0.25 ± 0.02c	0.51 ± 0.05ab	1.51 ± 0.05a	5.01 ± 0.07a
Vinegar processed	0.53 ± 0.01b	0.40 ± 0.03b	1.22 ± 0.08a	5.05 ± 0.03a
Wine processed	1.51 ± 0.01a	0.40 ± 0.03b	0.94 ± 0.07a	4.17 ± 0.29b
Pure compound	0.25 ± 0.01c	0.31 ± 0.02b	0.89 ± 0.09b	4.94 ± 0.12a
**Cerebrum**				
Crude extract	0.25 ± 0.00b	0.22 ± 0.24a	0.22 ± 0.03a	5.18 ± 0.10b
Vinegar processed	1.55 ± 0.03a	0.05 ± 0.01a	0.19 ± 0.03a	4.90 ± 0.10b
Wine processed	1.50 ± 0.01a	0.06 ± 0.01a	0.19 ± 0.04a	6.79 ± 0.34a
Pure compound	0.25 ± 0.02b	0.05 ± 0.01a	0.15 ± 0.03a	5.39 ± 0.19ab
**Cerebellum**				
Crude extract	0.25 ± 0.05c	0.07 ± 0.01a	0.29 ± 0.06a	6.16 ± 0.19a
Vinegar processed	1.09 ± 0.03a	0.06 ± 0.011a	0.20 ± 0.03ab	6.25 ± 0.30a
Wine processed	0.58 ± 0.08b	0.06 ± 0.01a	0.18 ± 0.04ab	6.82 ± 1.08a
Pure compound	0.25 ± 0.00c	0.05 ± 0.02a	0.17 ± 0.03b	5.67 ± 0.32a
**Diencephalons**				
Crude extract	2.12 ± 0.08a	0.07 ± 0.02a	0.35 ± 0.06a	9.22 ± 0.40a
Vinegar processed	1.07 ± 0.12b	0.09 ± 0.02a	0.26 ± 0.04ab	8.10 ± 0.29b
Wine processed	0.53 ± 0.04c	0.08 ± 0.02a	0.20 ± 0.04b	4.96 ± 0.15c
Pure compound	0.52 ± 0.11c	0.08 ± 0.02a	0.21 ± 0.05b	5.30 ± 0.12c
**Brain stem**				
Crude extract	0.51 ± 0.02b	0.22 ± 0.04a	0.60 ± 0.10a	4.21 ± 0.10c
Vinegar processed	1.53 ± 0.05a	0.17 ± 0.03a	0.58 ± 0.11a	6.25 ± 0.16a
Wine processed	0.54 ± 0.01b	0.17 ± 0.03a	0.50 ± 0.10a	5.46 ± 0.13b
Pure compound	0.50 ± 0.00b	0.16 ± 0.03a	0.41 ± 0.06a	4.29 ± 0.15c
**Hippocampus**				
Crude extract	1.51 ± 0.23b	0.09 ± 0.01a	0.37 ± 0.06a	8.15 ± 0.21a
Vinegar processed	1.54 ± 0.06b	0.07 ± 0.01a	0.28 ± 0.05a	8.68 ± 0.76a
Wine processed	2.07 ± 0.21a	0.07 ± 0.02a	0.28 ± 0.05a	9.35 ± 0.88a
Pure compound	1.08 ± 0.07c	0.07 ± 0.02a	0.27 ± 0.06a	8.43 ± 0.51a
**Striatum**				
Crude extract	0.25 ± 0.00c	0.09 ± 0.02a	0.20 ± 0.07a	7.15 ± 0.22a
Vinegar processed	1.01 ± 0.03a	0.06 ± 0.02a	0.24 ± 0.04a	6.70 ± 0.43a
Wine processed	0.52 ± 0.04b	0.06 ± 0.01a	0.22 ± 0.04a	5.19 ± 0.11b
Pure compound	0.25 ± 0.01c	0.06 ± 0.02a	0.21 ± 0.04a	7.27 ± 0.30a

Peak concentration of DHC in liver was the highest, followed by heart, lung, spleen, kidney and brain, respectively. In six parts of brain, C_max_ of DHC in brain stem and cerebrum was higher than other parts. No significant changes were observed on C_max_ and AUC_0–24__ h_ of DHC in all tissue examined after processing (*p* > 0.05). MRT of DHC ranged from 4.2 h (in heart) to 9.22 h (diencephalons) after administration of unprocessed *Rhizoma Corydalis*. Vinegar processing significantly prolonged the MRT of DHC in liver and brain stem while MRT of DHC in spleen, lung, cerebrum and diencephalons was shortened. The effect of wine processing on MRT of DHC in liver, kidney, cerebrum and striatum was different from the vinegar processing. In kidney and striatum, MRT of DHC was significantly decreased by wine processing of *Rhizoma Corydalis*, while no significant differences were observed in vinegar processing group. Wine processing significantly prolonged the MRT of DHC in cerebrum compared with crude extract group and vinegar processing group.

#### 2.1.4. Effect of Vinegar and Wine Processing on the Tissue Distribution of Protopine

**Figure 5:**
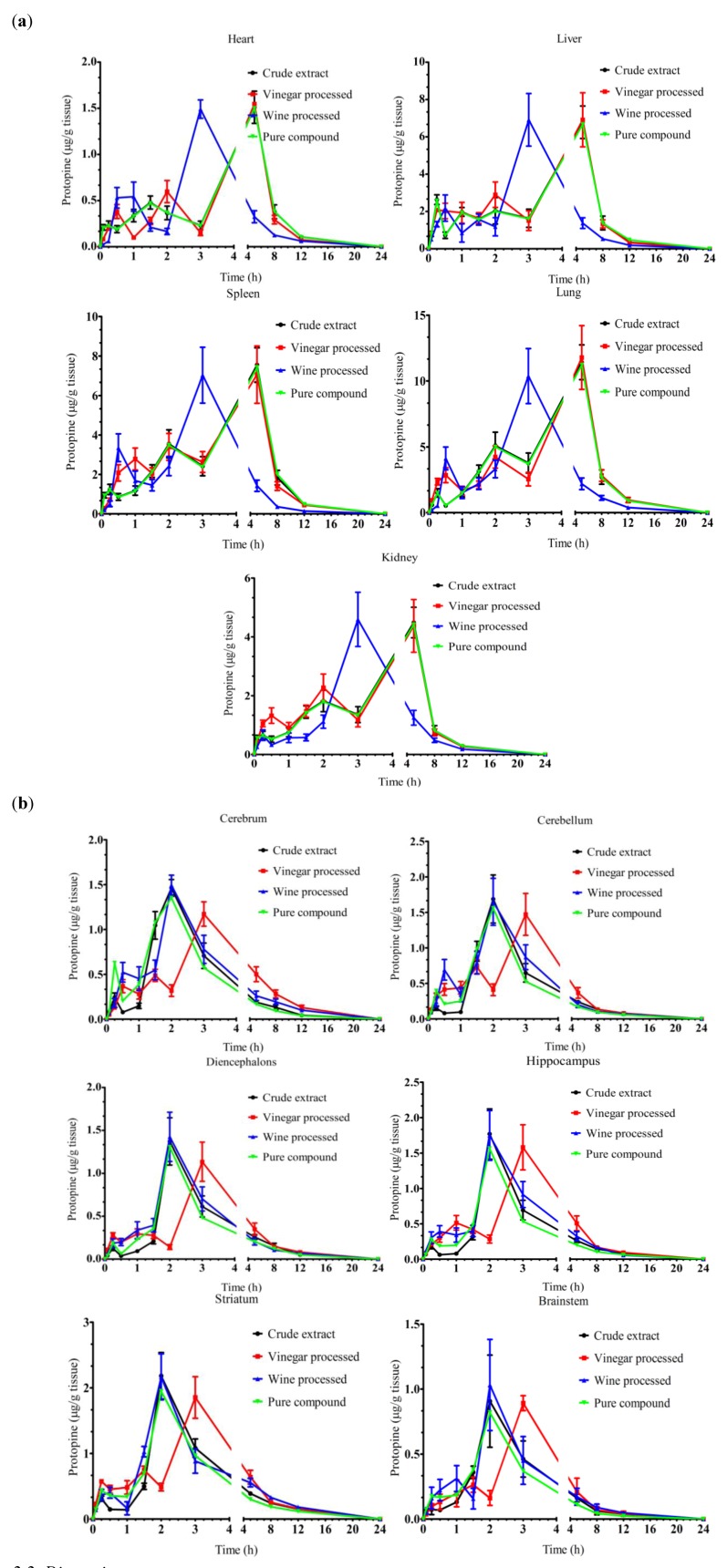
Time dependent changes of protopine in rat tissues. (**a**) Heart, liver, spleen, lung and kidney; (**b**) Six parts of brain including cerebrum, cerebellum, diencephalons, brainstem, hippocampus, striatum. Data are mean ± SD (n = 5).

Protopine reached the C_max_ at about 5 h in heart, liver, spleen, lung and kidney after administration of unprocessed *Rhizoma Corydalis* (Table 4 and Figure 5a). In six sections of brain, T_max_ of protopine was 2 h after administration of unprocessed *Rhizoma Corydalis*. Vinegar processing prolonged the T_max_ of protopine to 3 h in all six parts of brain (Figure 5b). In contrast, there was no significant change on T_max_ of protopine in heart, liver, spleen, lung and kidney after vinegar processing. Two hours decrease in the T_max_ of protopine were observed in wine processing group compared with unprocessed in the tissues of heart, liver, spleen, lung and kidney. However, wine processing did not change the T_max_ of propopine in brain. 

The highest C_max _of protopine among the tissues examined after administration of unprocessed of *Rhizoma Corydalis* was 11.42 ± 1.42 µg/mL in lung followed by spleen, liver, kidney, striatum, hippocampus, cerebellum, heart, cerebrum, diencephalons and brain stem. There were no significant differences on the value of C_max_ and AUC_0–24__ h_ of protopine after vinegar and wine processing. MRT of protopine was notably delayed by vinegar processing in lung, cerebrum, diencephalons, brain stem and hippocampus, while, it was significantly reduced in heart, spleen and kidney. Wine processing significantly shortened the MRT of protopine in heart, spleen, lung, kidney, cerebellum, hippocampus and diencephalons. 

**Table 4:** Pharmacokinetic parameters of protopine in rat tissues. Data are mean ± SD (n = 5). Data of four groups in each tissue indexed by different letters are significantly different according to one-way ANOVA followed by Tukey’s test (*p* < 0.05).

Tissue	T_max_ (h)	C_max _(µg/mL)	AUC_0–24__ h_ (µg·h/mL)	MRT (h)
**Heart**				
Crude extract	5.11 ± 0.24a	1.51 ± 0.18a	6.26 ± 0.92a	5.93 ± 0.02a
Vinegar processed	5.07 ± 0.15a	1.59 ± 0.43a	5.61 ± 1.06a	5.70 ± 0.03b
Wine processed	3.55 ± 0.31b	1.49 ± 0.30a	4.22 ± 0.85a	4.85 ± 0.03c
Pure compound	5.28 ± 0.42a	1.50 ± 0.45a	6.23 ± 1.56a	5.93 ± 0.02a
**Liver**				
Crude extract	5.32 ± 0.12a	6.78 ± 0.78a	29.16 ± 4.24a	5.46 ± 0.01b
Vinegar processed	5.25 ± 0.16a	6.91 ± 1.42a	28.13 ± 5.30a	5.28 ± 0.02b
Wine processed	3.07 ± 0.10b	6.91 ± 1.40a	19.30 ± 3.84a	5.46 ± 0.17ab
Pure compound	5.09 ± 0.68a	6.67 ± 1.97a	27.83 ± 6.98a	5.69 ± 0.03a
**Spleen**				
Crude extract	5.87 ± 0.32a	7.55 ± 0.87a	34.91 ± 5.19a	5.59 ± 0.03a
Vinegar processed	5.12 ± 0.34a	7.05 ± 1.45a	33.36 ± 6.42a	5.29 ± 0.04b
Wine processed	3.07 ± 0.61b	7.03 ± 1.41a	20.45 ± 4.13a	3.86 ± 0.03b
Pure compound	5.22 ± 0.05a	7.42 ± 2.22a	35.03 ± 8.18a	5.65 ± 0.03a
**Lung**				
Crude extract	5.14 ± 0.26a	11.42 ± 1.42a	51.16 ± 7.69a	5.86 ± 0.03b
Vinegar processed	5.63 ± 0.02a	11.78 ± 2.41a	50.69 ± 9.57a	5.97 ± 0.01a
Wine processed	3.87 ± 0.05b	10.38 ± 2.08a	31.54 ± 7.27a	4.76 ± 0.03c
Pure compound	5.22 ± 0.47a	11.22 ± 3.31a	50.24 ± 12.46a	5.85 ± 0.04b
**Kidney**				
Crude extract	5.01 ± 0.06a	4.49 ± 0.52a	18.65 ± 2.79a	5.54 ± 0.04a
Vinegar processed	5.09 ± 0.38a	4.37 ± 0.90a	18.48 ± 3.49a	5.32 ± 0.04b
Wine processed	3.10 ± 0.28b	4.60 ± 0.91a	13.78 ± 2.72a	4.93 ± 0.03c
Pure compound	5.13 ± 0.04a	4.42 ± 1.31a	13.44 ± 2.65a	5.53 ± 0.04a
**Cerebrum**				
Crude extract	2.09 ± 0.02b	1.46 ± 0.29a	3.87 ± 0.69a	4.22 ± 0.22b
Vinegar processed	3.11 ± 0.22a	1.15 ± 0.25a	5.02 ± 0.97a	6.37 ± 0.03a
Wine processed	2.08 ± 0.02b	1.50 ± 0.30a	4.57 ± 0.91a	6.21 ± 0.03a
Pure compound	2.10 ± 0.80b	1.35 ± 0.27a	3.65 ± 0.78a	3.93 ± 0.06b
**Cerebellum**				
Crude extract	2.13 ± 0.05b	1.69 ± 0.34a	4.41 ± 0.72a	5.56 ± 0.15a
Vinegar processed	3.28 ± 0.17a	1.47 ± 0.29a	4.93 ± 0.93a	4.94 ± 0.01b
Wine processed	2.21 ± 0.38b	1.65 ± 0.33a	4.67 ± 0.94a	4.98 ± 0.02b
Pure compound	2.08 ± 0.01b	1.56 ± 0.32a	3.80 ± 0.83a	4.86 ± 0.08b
**Diencephalons**				
Crude extract	2.11 ± 0.22b	1.37 ± 0.28a	3.48 ± 0.59a	4.94 ± 0.14c
Vinegar processed	3.08 ± 0.15a	1.14 ± 0.23a	3.98 ± 0.76a	5.76 ± 0.03a
Wine processed	2.03 ± 0.02b	1.43 ± 0.28a	3.91 ± 0.79a	5.42 ± 0.03b
Pure compound	2.07 ± 0.21b	1.31 ± 0.27a	3.18 ± 0.07a	4.89 ± 0.01c
**Brain stem**				
Crude extract	2.00 ± 0.08b	0.91 ± 0.19a	2.42 ± 0.46a	4.50 ± 0.18b
Vinegar processed	3.13 ± 0.41a	0.89 ± 0.18a	2.58 ± 0.46a	4.90 ± 0.19a
Wine processed	2.09 ± 0.10b	1.27 ± 0.21a	2.80 ± 0.57a	5.13 ± 0.03a
Pure compound	2.20 ± 0.01b	0.82 ± 0.17a	2.02 ± 0.42a	3.90 ± 0.14b
**Hippocampus**				
Crude extract	2.10 ± 0.03b	1.77 ± 0.36a	4.24 ± 0.87a	5.02 ± 0.15b
Vinegar processed	3.19 ± 0.18a	1.58 ± 0.31a	5.31 ± 0.97a	6.07 ± 0.16a
Wine processed	2.28 ± 0.01b	1.75 ± 0.35a	4.80 ± 0.96a	4.37 ± 0.03c
Pure compound	2.09 ± 0.04b	1.56 ± 0.31a	3.66 ± 0.81a	5.23 ± 0.10b
**Striatum**				
Crude extract	2.01 ± 0.01b	2.18 ± 0.44a	6.51 ± 1.44a	6.90 ± 0.20a
Vinegar processed	3.02 ± 0.07a	1.85 ± 0.37a	7.23 ± 1.29a	5.51 ± 0.26b
Wine processed	2.31 ± 0.02b	2.17 ± 0.44a	7.64 ± 1.50a	6.52 ± 0.01a
Pure compound	2.07 ± 0.08b	1.95 ± 0.40a	6.42 ± 1.52a	6.57 ± 0.43a

### 2.2. Discussion

The dried rhizome of *corydalis*, also called *Yanhusuo*, is a widely used traditional Chinese medicine. THP, DHC and protopine are three major bioactive components of the rhizome extract. Recently, Chen and the co-authors developed a HPLC-DAD method for simultaneous quantification of eight protoberberine quaternary alkaloids from ethanol extract of *Rhizoma corydalis* [19]. A HPLC-ESI/MS method was reported for simultaneous determination of THP, protopine and palmatine in rat plasma in 2009 [20]. However, there are still no reports on simultaneous measurement of the three main bioactive compounds, namely THP, DHC and protopine, in animal tissues after administration of rhizome extract. In this study, we developed and validated a simple, accurate and sensitive method for the simultaneous determination of THP, DHC and protopine in rat tissues by HPLC-UV.

Processing is a crucial step before the Chinese medicinal herbs can be used in prescriptions. Two processed products of *Rhizoma corydalis* (vinegar and wine) have been clinically used in China for thousands of years based on the Traditional Chinese Medicine (TCM) Theory [21]. THP is the major bioactive compound with analgesic effect [4]. It only took 15 min for THP to reach the C_max_ in all the tissues examined after administration of unprocessed *Rhizoma corydalis* (Figure 3). This indicated that *Rhizoma corydalis* is a fast pain relief drug. Vinegar processed *Rhizoma corydalis* is recommended in TCM prescriptions when they are used for the treatment of the pain in stomach, chest and dysmenorrhe [22]. Previous report showed that the analgesic effect of *Rhizoma corydalis* was more intense when vinegar processed products were used compared with unprocessed *Rhizoma corydalis* [17]. However, in this study, we observed that vinegar processing does not significantly change the tissue distribution of THP. The improvement of analgesic effect by using vinegar processed *Rhizoma corydalis* might be because of the increase of THP when the same amount of plant materials was used [18]. The slower absorption of THP was observed after wine processing in heart, liver, spleen, lung and cerebrum. Therefore, wine processed products should not be used for the treatment of acute pain in these organs. In addition, several THP poisoning cases were reported after administration of THP pure compound [23]. In our study, the concentration of THP in the tissues dropped quickly after it arrived at the peak concentration and after 12 h, the concentration of THP in the tissues was only 1/17~1/49 of the C_max_. This indicated that administration of *Rhizoma corydalis* instead of THP pure compound will not cause the accumulation of THP in the tissue.

DHC is the bioactive compound in *Rhizoma corydalis* for the treatment of coronary heart disease through the protective effect on cardiomyocytes and the inhibition of myocardial apoptosis [9,10,24]. Our results supported this conclusion because the C_max_ of DHC was higher in heart than that in other tissues except liver after administration of unprocessed *Corydalis* (Table 3). Wine processed *Rhizoma corydalis* is widely used in TCM practices for coronary heart diseases [25]. However, in our study,both vinegar and wine processing deferred the time for DHC to reach the peak concentration in heart. In addition, there were no significant changes on C_max_ and AUC_0–24__ h_. These results indicated that processing is not necessary when *Rhizoma corydalis* is used for the treatment of coronary heart disease.

It is well known that protopine has vasodilator effects which are related to the elevations of cAMP and cGMP and calcium antagonism [26]. Our data showed that C_max_ of protopine in lung is higher than in other tissues after administration of unprocessed *Rhizoma corydalis* (Table 4). This indicated that *Rhizoma corydalis* has better vasodilator effect on the smooth muscle in lung and can be used for the treatment of asthma. Wine processing accelerated the distribution of protopine in lung by reducing the T_max_, in contrast, processing of *Rhizoma corydalis* with vinegar significantly increased the MRT of protopine in lung without changing other pharmacokinetic parameters. Therefore, wine processing is recommended when *Rhizoma corydalis* is used for cough relief. In addition, recent studies showed that protopine is able to relieve H_2_O_2_-induced oxidative stress and apoptosis caused by cerebral ischaemic [11,27]. In this study, we found that the speed of the tissue distribution of protopine was much slower than the unprocessed group in all six sections of the brain (Table 4). A possible reason is that the acetic acid in vinegar forms a complex with protopine and the diffusion of the protopine-acetic acid salt to the brain tissues might be restricted by the blood brain barrier [28]. Based on our results, we suggested that either unprocessed or processed with wine should be used for cerebral ischaemia. 

## 3. Experimental

### 3.1. Chemicals

THP, protopine and nuciferine pure compound were obtained from the National Institute for the Control of Pharmaceutical and Biological Products (Beijing, China). DHC was obtained from Wako Pure Chemical Industries, LTD. (Japan). Unprocessed *Rhizoma Corydalis* was purchased from Tianjin ZhongXin Pharmaceutical Group Corporation Limited (Tianjin, China). HPLC-grade methanol and acetonitrile were purchased from Tianjin Union Hope Chromatography Technological Corporation Limited (Tianjin, China). Yellow wine and edible vinegar used for processing were purchased from Tianjin Tianliduliu Mature Vinegar Co. LTD. The content of ethanol in the wine is 15%–20% (v/v). The vinegar is derived from rice and the acetic acid content in the vinegar is 4%–6% (v/v).

### 3.2. Animals

The experimental protocol was approved by the University’s Animal Ethics Committee. Healthy Wistar male rats (body weight 250 g ± 20 g, mean ± SD) were purchased from Institute of Laboratory Animal Sciences, Chinese Academy of Medical Sciences (Beijing, China) and maintained in a temperature and humidity controlled facility. Rats had unlimited access to water. Standard food was given until 12 h before the experiments.

### 3.3. Preparation of *Rhizoma Corydalis* Extract

*Rhizoma Corydalis* was processed according to Pharmacopoeia of Chinese Medicine and National Guideline of Traditional Chinese Medicinal Plants Processing [12]. Briefly, fresh *Rhizoma Corydalis* was boiled in water till the pith turn light brown color. Cooked rhizome was sliced into pieces (4 mm, diameter) and dried at 25 °C. Dried slices were mixed with edible vinegar (100:20, rhizome/vinegar, w/v) or yellow wine (100:10, rhizome/wine, w/v) and the slices allowed to completely absorb the vinegar or wine. The soaked slices were then stir-fried in a metallic pan over a low flame till they were completely dry. The processed *Rhizoma Corydalis* was added with 70% ethanol (1:30 w/v) and sonicated for 1 h at room temperature. The extract was then concentrated to remove the ethanol residue and stored at 4 °C for further use. The major components in the ethanol extract are three alkaloids including THP, DHC and protopine [15]. The ethanol extract of the unprocessed *Rhizoma Corydalis* and THP, DHC and protopine pure compound were used as the controls.

### 3.4. Administration and Tissue Sample Collection

Three hundred and sixty healthy rats were used in total and are divided into six groups (Group 1–6, sixty rats per group). Drugs were administrated by gastric gavage method. In order to keep the dose of THP, DHC and protopine constant in each group, the amount of ethanol extract of *Rhizoma Corydalis* administrated was normalized based on the dose of protopine pure compound used. The dose of THP and DHC pure compound administrated was same with that in the ethanol extract of *Rhizoma Corydalis*. The details were listed below. Group 1: Rats were administrated with the 70% ethanol extract of unprocessed *Rhizoma Corydalis* at a dose of 17.75 g extract/kg body weight. Group 2: Rats received the extract of vinegar-processed *Rhizoma Corydalis* at a dose of 15 g extract/kg rat body weight. Group 3: Rats were administrated with the extract of wine processed *Rhizoma Corydalis* at a dose of 15.78 g extract/kg body weight. Group 4: Rats were administrated 4.52 mg/kg body weight of protopine pure compound. Group 5: Rats received THP pure compound at a dose of 6.45 mg/kg body weight. Group 6: Rats were administrated 26.10 mg of DHC pure compound/kg body weight. Blood and tissues (heart, liver, spleen, lung, kidney, brain) were collected at 0.083, 0.25, 0.5, 1, 1.5, 2, 3, 5, 8, 12 h (5 rats/per group/per time) after administration. Brian tissue was further dissected into six parts: cerebrum, cerebellum, diencephalons, brain stem, hippocampus and striatum. All the tissue samples were rinsed with ice-old saline and stored at −20 °C until extraction.

### 3.5. Extraction Procedures

Tissue samples were homogenized in three volumes of ice-cold biological saline and centrifuged for 10 min at 10,000 g and 4 °C. An aliquot of the supernatant (0.5 mL) was collected. Liquid-liquid extraction was used to extract THP, DHC and protopine. One hundred microliter of 1.0 µg/mL nuciferin (internal standard) was mixed with 0.5 mL aliquots of the supernatant. The mixture was extracted with 1.49 mL of methanol by vortexing for 2 min and centrifuged at 10,000 g for 10 min. The supernatant was evaporated completely under the nitrogen stream in a 60 °C water bath. The residue was redissolved in 100 µL of methanol.

### 3.6. Simultaneous Determination of THP, DHC and Protopine in Rat Tissues

A CoMetro HPLC system (CoMetro Technology Ltd, South Plainfiled, NJ, USA) with 6000 PVW detector UV-VIS detector was used. Separation was achieved by injecting 50 µL sample into a Kromasil ODS-C_18_ (250 mm × 4.6 mm, 5 µm) (packed by Dalian Elite Analytical Instruments Co. Ltd., Liaoning, China). Other chromatographic conditions were: column temperature, 35 °C; mobile phase, acetonitrile-0.1% phosphoric acid (pH 3.0) (30:70 v/v); flow rate, 1 mL/min; wavelength, 280 nm.

### 3.7. Validation of the Method for Quantitative Analysis of THP, DHC and Protopine in Rat Tissues

Method validation was examined for assay specificity, precision, linearity, accuracy, and extraction recovery under the HPLC analytical conditions described above. Stock solution of THP, protopine, and DHC was prepared in methanol at concentration of 150 µg/mL and mixed. Working solution of the analytes was prepared by serial dilution of the stock. The specificity of the method was assessed by preparing and analyzing six different batches of drug-free rat tissues. The chromatograms of blank tissue samples were compared with those obtained with the analytes (THP (0.306 µg/mL), DHC (0.294 µg/mL), protopine (0.320 µg/mL) and nuciferine (1.0 µg/mL)) and those after administration of *Rhizoma Corydalis* extracts. The standard curve was constructed using a linear least-square regression equation derived from the peak area. Accuracy was evaluated as the relative error (R.E.), and precision was determined as the relative standard deviation (R.S.D.). The accuracy and the precision of the assays for intra-day and inter-day determinations were evaluated by the analysis of three concentrations (six replicates for each) on the same day and on three consecutive validation days, respectively. The relative extraction recovery was calculated by comparing the peak area of tissue-extracted standards with that obtained from the extracted blank tissue sample post spiked with the corresponding standards (n = 5). The absolute recovery was calculated by comparing the peak area of extracted samples with that of the standard solution containing an equivalent amount of the analytes (n = 5).

### 3.8. Pharmacokinetic and Statistical Analysis

Pharmacokinetic parameters were estimated by non-compartmental analysis using Equiv Test/PK (Statistical Solutions Ltd., Saugus, MA, USA). The maximum concentration (C_max_) and the time to reach the maximum concentration (T_max_) were directly obtained from the observed value. All data were represented as mean ± SD. Comparison of values between groups was performed using one-way ANOVA followed by Tukey’s test for comparisons among four groups. A value of *p* < 0.05 was considered significantly difference.

## 4. Conclusions

In this paper, a sensitive, simple and accurate HPLC method has been developed and validated for the simultaneous quantification of the three main bioactive compounds (THP, DHC and protopine) in rat tissues after administration of ethanol extract of *Rhizoma corydalis*. The validated method has been successfully used to investigate the effect of vinegar and wine processing on the distribution of THP, DHC and protopine in the rat tissues. Our results showed that vinegar and wine processing mainly affect the T_max_ and MRT and the effect varies in different tissues and among different bioactive compounds. Our results suggested that differently processed *Rhizoma corydalis* products (unprocessed, wine processed or vinegar processed) should be selected according to the specific disease condition and the affected organs.
